# Differential diagnosis of benign and malignant patchy ground-glass opacity by thin-section computed tomography

**DOI:** 10.1186/s12885-022-10338-4

**Published:** 2022-11-23

**Authors:** Zhang-rui Liang, Min Ye, Fa-jin Lv, Bin-jie Fu, Rui-yu Lin, Wang-jia Li, Zhi-gang Chu

**Affiliations:** Department of Radiology, The First Affiliated Hospital of Chongqing Medical University, 1# Youyi Road, Yuanjiagang, Yuzhong district, Chongqing, China

**Keywords:** Benign, Ground-glass opacity, Malignancy, X-ray computed, Tomography

## Abstract

**Background:**

Previous studies confirmed that ground-glass nodules (GGNs) with certain CT manifestations had a higher probability of malignancy. However, differentiating patchy ground-glass opacities (GGOs) and GGNs has not been discussed solely. This study aimed to investigate the differences between the CT features of benign and malignant patchy GGOs to improve the differential diagnosis.

**Methods:**

From January 2016 to September 2021, 226 patients with 247 patchy GGOs (103 benign and 144 malignant) confirmed by postoperative pathological examination or follow-up were retrospectively enrolled. Their clinical and CT data were reviewed, and their CT features were compared. A binary logistic regression analysis was performed to reveal the predictors of malignancy.

**Results:**

Compared to patients with benign patchy GGOs, malignant cases were older (*P* <  0.001), had a lower incidence of malignant tumor history (*P* = 0.003), and more commonly occurred in females (*P* = 0.012). Based on CT images, there were significant differences in the location, distribution, density pattern, internal bronchial changes, and boundary between malignant and benign GGOs (*P* <  0.05). The binary logistic regression analysis revealed that the independent predictors of malignant GGOs were the following: patient age ≥ 58 years [odds ratio (OR), 2.175; 95% confidence interval (CI), 1.135–6.496; *P* = 0.025], locating in the upper lobe (OR, 5.481; 95%CI, 2.027–14.818; *P* = 0.001), distributing along the bronchovascular bundles (OR, 12.770; 95%CI, 4.062–40.145; *P <* 0.001), centrally distributed solid component (OR, 3.024; 95%CI, 1.124–8.133; *P* = 0.028), and well-defined boundary (OR, 5.094; 95%CI, 2.079–12.482; *P <* 0.001).

**Conclusions:**

In older patients (≥58 years), well-defined patchy GGOs with centric solid component, locating in the upper lobe, and distributing along the bronchovascular bundles should be highly suspected as malignancy.

## Background

With the advancements in computed tomography (CT) and the initiation of CT screening for lung cancer, pulmonary nodules, particularly those with ground-glass opacities (GGOs), are now frequently detected [1–6]. GGOs are defined as areas with a slight homogeneous increase in density that does not obscure the underlying bronchial structures or vascular margins on high-resolution CT (HRCT) [7]. Based on the presence of solid components, GGOs can be further divided into mixed ground-glass opacities (mGGOs) and pure ground-glass opacities (pGGOs) [8, 9]. The nature of GGOs is diverse; it may be caused by benign disorders (pulmonary interstitial thickening, edema, fibrosis, partial alveolar collapse, normal breathing state, increased blood volume of capillaries from the incompletely filled alveolar cavity, or inflammation) or pulmonary adenocarcinoma [2, 7, 10–13]. Thus, the differential diagnosis of GGOs is frequently required in clinical practice.

GGOs usually manifest as round, oval, or irregular nodules (ground-glass nodules, GGNs) or patches on CT images. The differentiation of malignant and benign GGNs has been the focus of radiological studies [9, 14–20]. Previous studies confirmed that GGNs with certain CT manifestations, such as larger size, higher density, lobulation, spiculation, pleural indentation, vacuole sign, well-defined border, or vascular convergence, had a higher probability of malignancy [14, 16–18, 20]. Similar to GGNs, patchy GGOs may also be benign or malignant. Moreover, pulmonary inflammation frequently appears as patchy opacities; thus, neoplastic GGOs may be misdiagnosed as inflammation, and the optimal curative opportunity is missed. Therefore, distinguishing benign patchy GGOs from malignant ones is of great significance. However, until now, differentiating these two kinds of lesions has not been discussed solely [5, 6, 8, 9, 19, 21, 22].

In view of the lack of experience in differentiating benign from malignant patchy GGOs and the possible different CT manifestations of patchy GGOs and GGNs, this study aimed to investigate the clinical and CT characteristics of patchy GGOs and clarify the distinct CT features of benign and malignant ones to improve their diagnosis and differential diagnosis.

## Methods

### Study design and patient enrollment

From January 2016 to September 2021, patients with patchy GGOs confirmed by postoperative pathological examination or follow-up (lesions that decreased significantly in size or disappeared on repeated CT images) were enrolled. Patchy GGOs were defined as GGOs without a specific shape (round or oval) and cannot be described as nodules or mass. The inclusion criteria were as follows: patients with patchy GGOs and patients with complete clinical and imaging data. The exclusion criteria were as follows: CT images with a thickness > 1 mm and CT images with severe artifacts affecting the evaluation. Finally, 139 patients with 144 malignant GGOs and 87 patients with 103 benign GGOs were included in this study.

### Ethics approval and consent to participate

This study conformed to the Declaration of Helsinki on Human Research Ethics standards and was approved by the institutional review board of the First Affiliated Hospital of Chongqing Medical University (number 2019–062). The need for written, informed consent was waived by the institutional review board of the First Affiliated Hospital of Chongqing Medical University because of the retrospective design.

### CT examinations

CT imaging was performed using one of the following scanners: SOMATOM Perspective (Siemens Healthineers, Erlangen, Germany), SOMATOM Definition Flash (Siemens Healthineers, Erlangen, Germany), or Discovery CT750 HD (GE Healthcare, Milwaukee, WI, USA). The patients were placed in a supine position with their arms lifted upward and the head closed to the scanner. CT scans were performed at the end of inspiration during a single breath-hold. The scan range was from the tip to the base of the lungs, including the bilateral chest wall and axillae. The scanning parameters were as follows: tube voltage, 110–130 kVp; tube current time, 50–140 mA (using automatic current modulation technology); scanning slice thickness, 5 mm; rotation time, 0.5 s; pitch, 1–1.1; collimation, 0.6 or 0.625 mm; reconstruction slice thickness and interval, 0.625 or 1 mm; and matrix, 512 × 512. All images were reconstructed with 0.625 or 1 mm slice thickness using a standard algorithm or medium-sharp algorithm.

### Clinical data

The patients’ clinical data were recorded using the Electronic Medical Record System (Winning Health, China). Clinical data, including the patients’ age, sex, smoking history, history of cancer, family history of lung cancer, and respiratory symptoms (cough, expectoration, chest distress, chest pain, and hemoptysis), were recorded.

### Image analysis

All CT images were viewed in lung and mediastinal window settings. Multiplanar reconstructions (MPRs) were performed to display the morphological features of the lesions. The CT images of all patients were reviewed by two radiologists (with 20 and 15 years of experience in chest CT) who were blinded to the clinical data and results. Any disagreements during review were resolved by consensus.

**Fig. 1:**
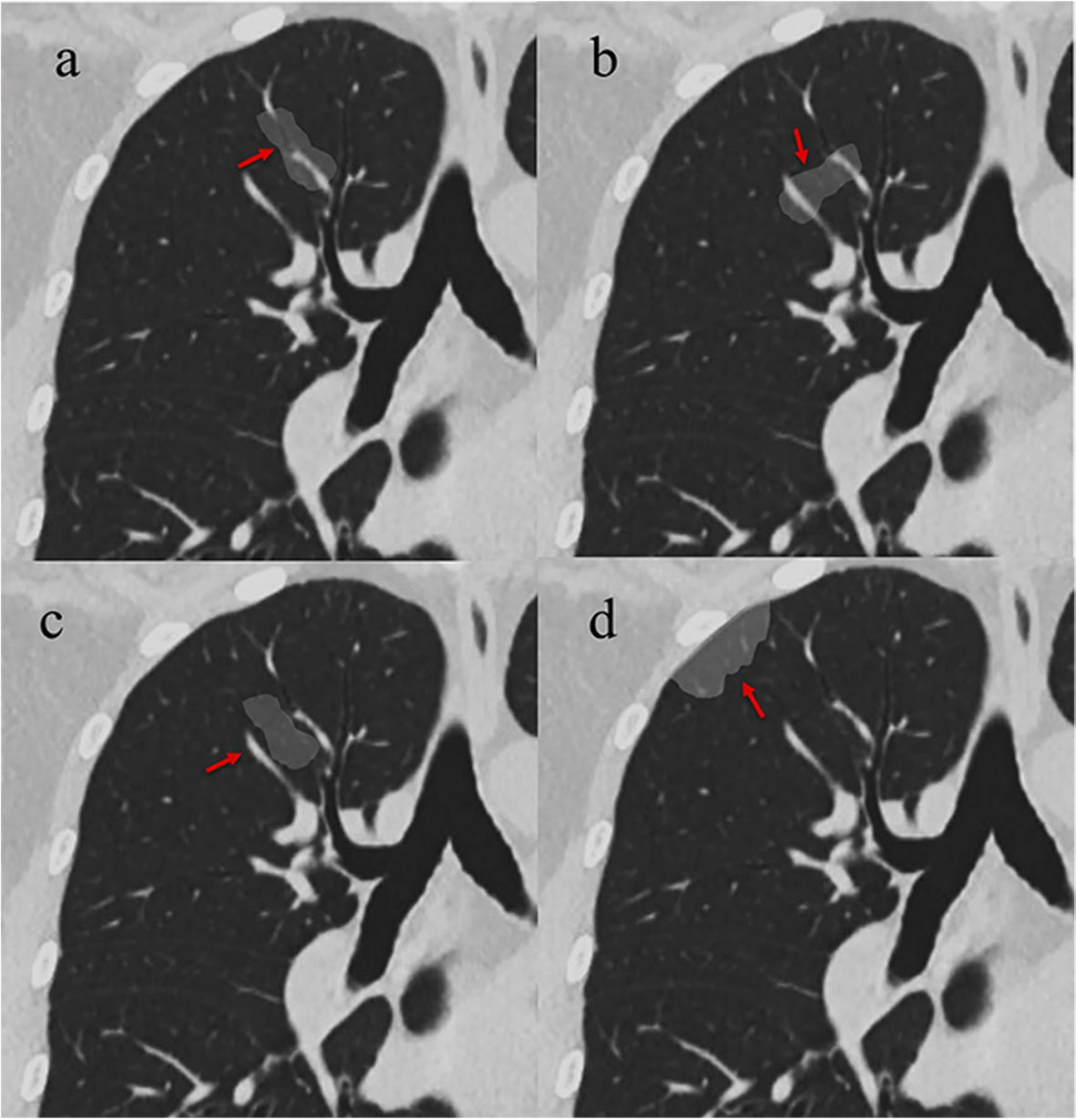
Distribution of GGOs in relation to the bronchovascular bundles. **a** type I, the lesion surrounds the bronchi and blood vessels, and its long axis is consistent with their direction. **b** type II, the lesion surrounds the bronchi and blood vessels, but its long axis crosses them. **c** type III, the lesion is located among the bronchovascular bundles, the surrounding bronchi and blood vessels may be partly involved or not. **d** type IV, the lesion is mainly located in the subpleural zone. GGOs, ground-glass opacities

The overall CT features of each patchy GGO were analyzed: (a) size (the mean of the longest diameter and perpendicular diameter on MPR images), (b) density pattern (pGGO, mGGO with centrally distributed solid component, and mGGO with scattered solid component), (c) location (upper lobe, middle lobe, or lower lobe), (d) boundary (well-defined or ill-defined), (e) dilated internal bronchus (yes or no), and (f) distribution in relation to the bronchovascular bundles. The lesion distribution in relation to the bronchovascular bundles was classified into four types based on MPR images: type I, the lesion distributed along the bronchovascular bundles; type II, the lesion crossed the bronchovascular bundles; type III, the lesion distributed among the bronchovascular bundles; type IV, the lesion distributed in the subpleural zone (Fig. 1). If the lesion mainly involved the subpleural lung tissue, it was classified as type IV regardless of the presence of surrounding peripheral vessels. An ill-defined boundary was considered when the lesion’s periphery faded out into the adjacent normal lung parenchyma and the junction between the lesion and surrounding lung parenchyma could not be clearly defined. If the diameter of the bronchus within the lesions was equal or larger to that of the normal proximal segment, it was considered dilated.

### Statistical analysis

Statistical analysis was performed using SPSS software (version 24, IBM Corp., Armonk, NY). Continuous data (patients’ age, lesion size) are expressed as mean ± standard deviation, whereas categorical variables are presented as numbers and percentages. Shapiro-Wilk test was used to determine if the variables followed a normal distribution. Continuous data were analyzed using Mann-Whitney U test (patients’ age, lesion size). Categorical data were analyzed using Pearson’ s χ^2^ test (patients’ sex, smoking history, history of cancer, family history of lung cancer, respiratory symptoms, density pattern, boundary, internal bronchial changes, and distribution in relation to the bronchovascular bundles) or Fisher’s exact test (lesion location). Statistical significance was accepted at *P* <  0.05.

For variables with statistical significance, a binary logistic regression analysis was applied to estimate the likelihood of malignancy; *P* ≤ 0.05 was considered as the criteria for variable inclusion. The patients’ age was dichotomized at a cut-off value of 58 years, which was determined by receiver operator characteristics curve analysis (ROC). ROC analysis was also conducted for a binary logistic regression model. The optimal cut-off values were determined as the point closest to the upper left-hand corner of the ROC curve.

## Results

### Patients’ clinical characteristics

The patients’ clinical data are summarized in Table 1. Patients with malignant GGOs (61.5 ± 9.5 years) were older than those with benign ones (52.6 ± 12.7 years, *P* <  0.001). Female (59.7% vs. 42.5%, *P* = 0.012) and individuals without malignant tumor history (93.5% vs. 80.5%, *P* = 0.003) were more common in patients with malignant GGOs than in those with benign ones.

**Table 1:** Comparison of the patients’ clinical characteristics

Clinical features	Patients with malignant GGOs (***n*** = 139)	Patients with benign GGOs (***n*** = 87)	***P*** Value
Age (years)	61.5 ± 9.5	52.6 ± 12.7	< 0.001
Gender			
Female	83 (59.7)	37 (42.5)	0.012
Male	56 (40.3)	50 (57.5)	
Smoking history			
Yes	42 (30.2)	27 (31.0)	0.897
No	97 (69.8)	60 (69.0)	
History of malignant tumor			
Yes	9 (6.5)	17 (19.5)	0.003
No	130 (93.5)	70 (80.5)	
Family history of lung cancer			
Yes	11 (7.9)	1 (1.1)	0.057
No	128 (92.1)	86 (98.9)	
Respiratory symptoms			
Yes	34 (24.5)	14 (16.1)	0.134
No	105 (75.5)	73 (83.9)	

Data are expressed as number (percentage) or mean ± standard deviation

*GGOs* ground-glass opacities

### CT features of benign and malignant patchy GGOs

The CT findings of benign and malignant patchy GGOs are shown in Table 2. There were significant differences in the size of atypical adenomatous hyperplasia (AAH)- adenocarcinoma in situ (AIS) group, minimally invasive adenocarcinoma (MIA) group, and invasive adenocarcinomas (IAC) group (*P* <  0.001). Compared with the AAH-AIS group and MIA group, the GGOs in the IAC group were larger (22.6 ± 7.7 mm vs. 13.1 ± 3.3 mm, *P* <  0.001; 22.6 ± 7.7 mm vs. 15.8 ± 4.4 mm, *P* <  0.001). Compared with benign GGOs, malignant GGOs were more located in the upper lobe (*P* <  0.001) and distributed along the bronchovascular bundles (*P* < 0.001). With respect to lesions, more malignant GGOs had centric solid component (*P* < 0.001), dilated internal bronchus (*P* < 0.001), and well-defined boundary (*P* < 0.001).

**Table 2:** Comparison of CT features of malignant and benign GGOs

CT features	Malignant GGOs (***n*** = 144)	Benign GGOs (***n*** = 103)	***P*** Value
Diameter (mm)	20.3 ± 7.6	21.1 ± 11.6	0.428
Location			
Upper lobe	115 (79.9)	53 (51.5)	< 0.001
Middle lobe	6 (4.2)	4 (3.9)	
Lower lobe	23 (16.0)	46 (44.7)	
Density pattern			
pGGO	22 (15.3)	53 (51.5)	< 0.001
mGGO with centric solid component	89 (61.8)	27 (26.2)	
mGGO with scattered solid component	33 (22.9)	23 (22.3)	
Boundary			
Well-defined	96 (66.7)	29 (28.2)	< 0.001
Ill-defined	48 (33.3)	74 (71.8)	
Internal dilated bronchus			
Yes	89 (61.8)	13 (12.6)	< 0.001
No	55 (38.2)	90 (87.4)	
Distribution in relation to bronchovascular bundles			
Type I	122 (84.7)	18 (17.5)	< 0.001
Type II	2 (1.4)	25 (24.3)	
Type III	8 (5.6)	25 (24.3)	
Type IV	12 (8.3)	35 (34.0)	

Data are expressed as number (percentage) or mean ± standard deviation

*CT* computed tomography, *GGOs* ground-glass opacities, *pGGO* pure ground-glass opacity, *mGGO* mixed ground-glass opacity

### Binary logistic regression analysis and ROC analysis

**Fig. 2:**
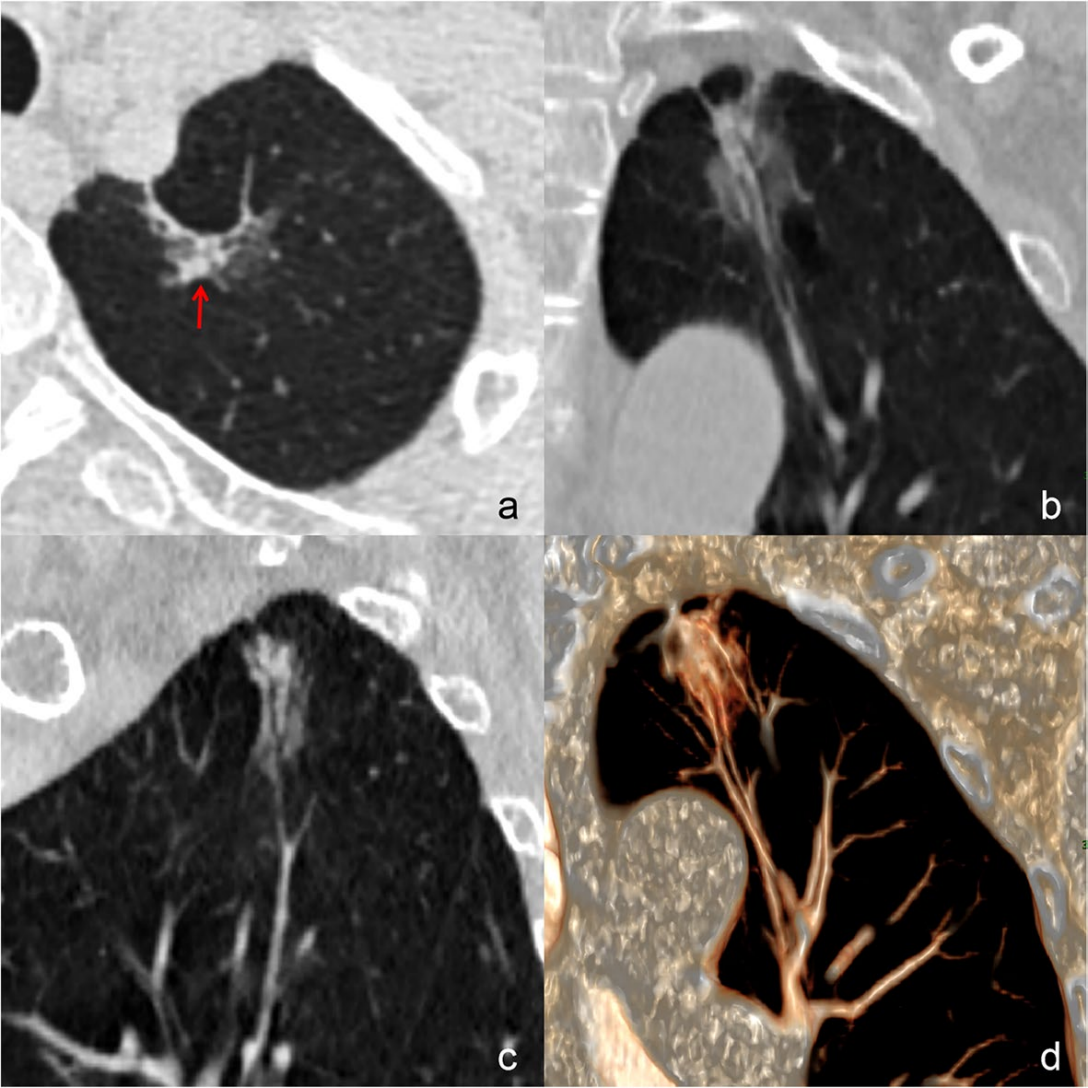
A 67-year-old female with invasive adenocarcinoma. **a** A well-defined patchy mGGO locates in the left upper lobe. The irregular solid component (red arrow) is surrounded by GGO. Coronal (**b**) and sagittal (**c**) images show that the lesion surrounds the bronchi and blood vessels, and its long axis is consistent with their direction. The lumen of internal bronchi is dilated. **d** VR image shows that the lesion distributes along the blood vessels. GGO, ground-glass opacity; mGGO, mixed ground-glass opacity; VR, volume rendering

**Fig. 3:**
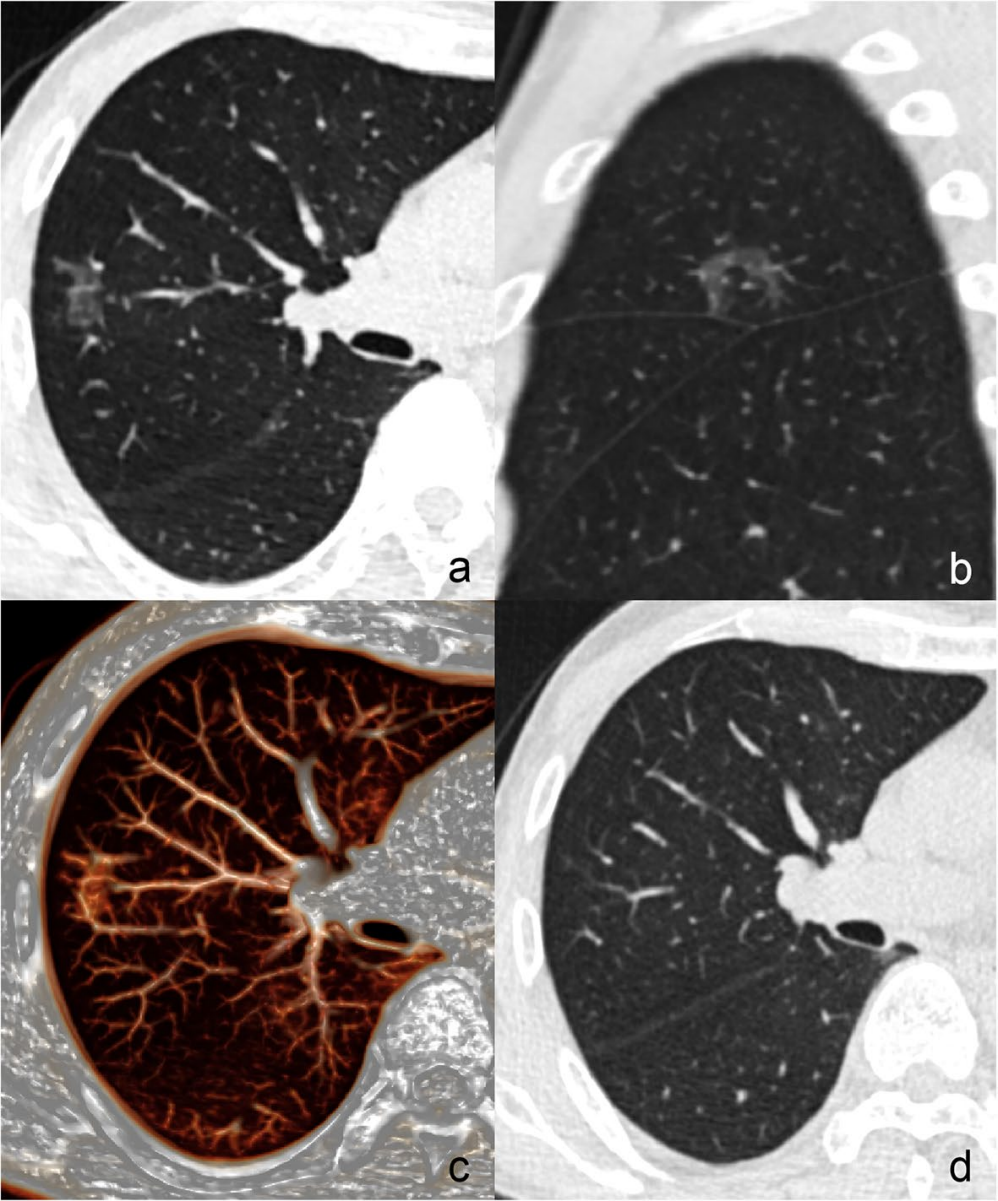
A 56-year-old male with benign patchy GGO. **a** A well-defined patchy pGGO locates in the right upper lobe. Axial (**a**) and sagittal (**b**) images show that the lesion surrounds the blood vessels, but its long axis crosses them. The dilated internal bronchus is not seen. **c** VR image shows that the lesion crosses the blood vessels. **d** The lesion disappears after 10 months. GGO, ground-glass opacity; pGGO, pure ground-glass opacity; VR, volume rendering

**Fig. 4:**
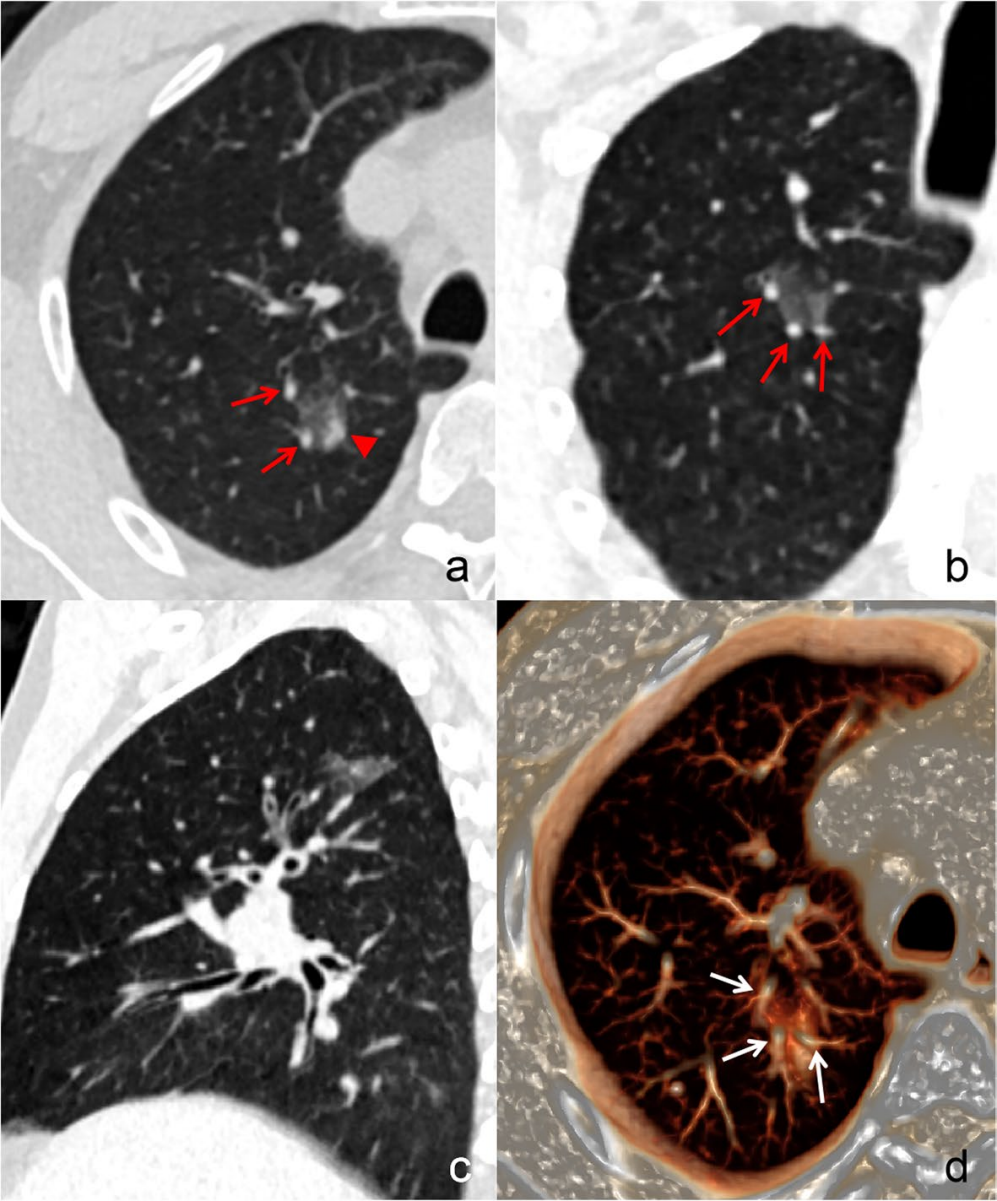
A 55-year-old male with benign patchy GGO. Axial (**a**) and oblique coronal (**b**) images show that an ill-defined patchy mGGO with eccentric solid component (red arrow head) locates in the right upper lobe. The adjacent blood vessels (red arrows) are partly involved. Sagittal (**c**) image shows that the lesion does not surround the bronchi or blood vessels. **d** VR image shows that the lesion is located among the blood vessels (white arrows). GGO, ground-glass opacity; mGGO, mixed ground-glass opacity; VR, volume rendering

**Fig. 5:**
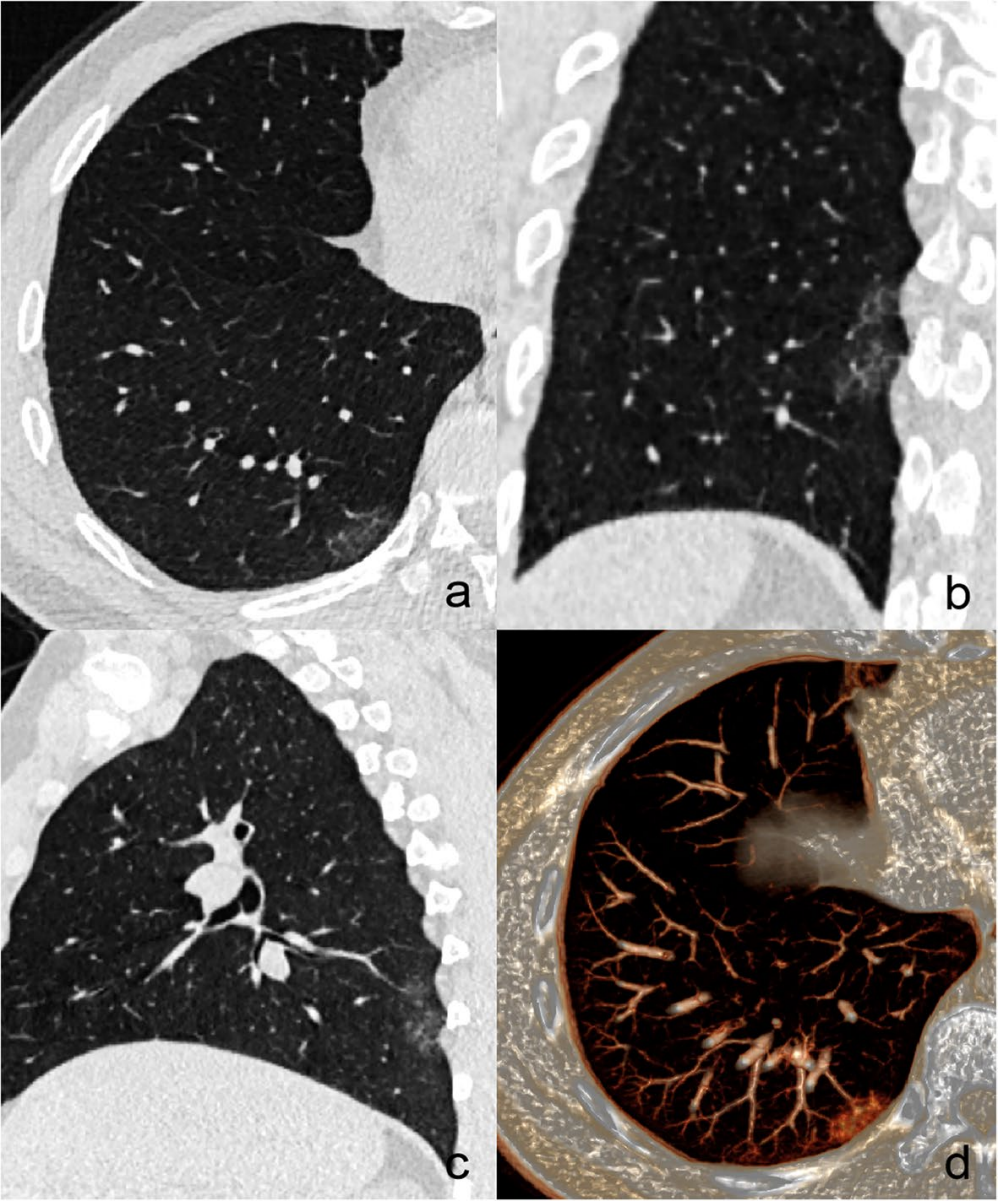
A 41-year-old male with benign patchy GGO. **a** An ill-defined patchy pGGO locates in the subpleural zone of the right lower lobe. Multiple peripheral vessels can be detected in it. Coronal (**b**) and sagittal (**c**) images show that the lesion has a wide base. **d** VR image shows that the lesion is located in the subpleural zone, the adjacent big vessels are not involved. GGO, ground-glass opacity; pGGO, pure ground-glass opacity; VR, volume rendering

Binary logistic regression analysis revealed that the patients’ age ≥ 58 years [odds ratio (OR), 2.175; 95% confidence interval (CI), 1.135–6.496; *P* = 0.025], location in the upper lobe (OR, 5.481; 95%CI, 2.027–14.818; *P* = 0.001), distribution along bronchovascular bundles (OR, 12.770; 95%CI, 4.062–40.145; *P* < 0.001), centrally distributed solid component (OR, 3.024; 95%CI, 1.124–8.133; *P* = 0.028), and well-defined boundary (OR, 5.094; 95%CI, 2.079–12.482; *P* < 0.001) were independent predictors of malignant GGOs (Figs. 2, 3, 4 and 5).

**Fig. 6:**
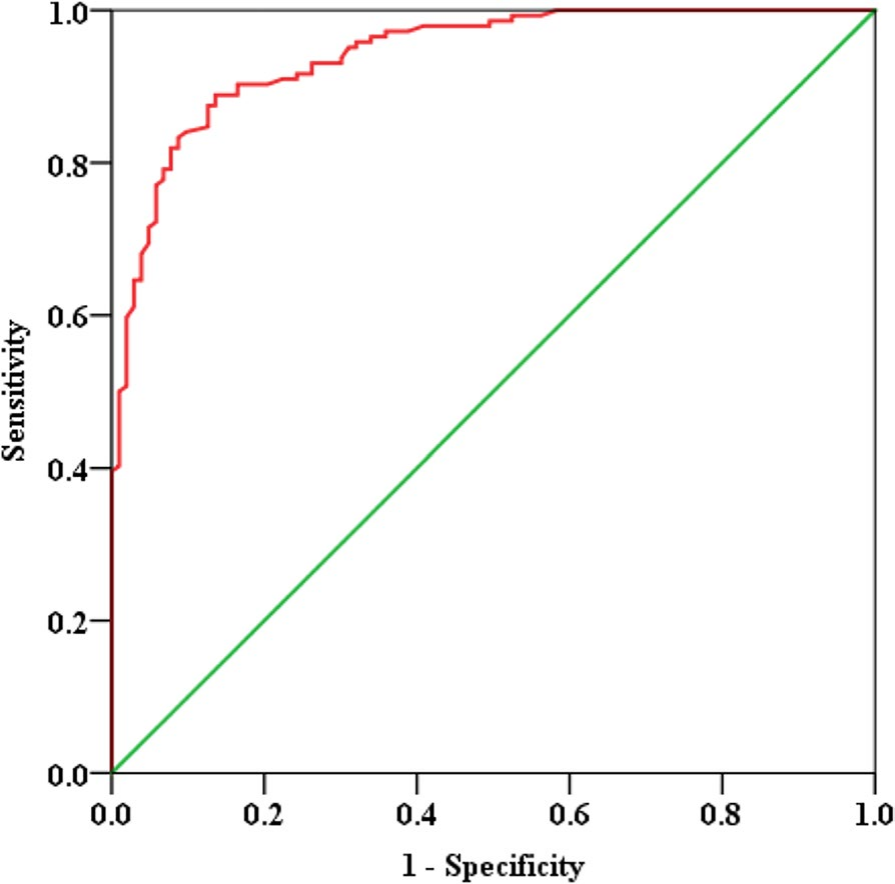
Graph shows the result of logistic regression model in discriminating malignant GGOs from benign ones. GGOs, ground-glass opacites

ROC analysis was performed to evaluate the performance of the binary logistic regression models in discriminating benign GGOs from malignant ones. The area under the ROC curve was 0.942 (95%CI, 0.915–0.968; *P* < 0.001), and the sensitivity and specificity of this model were 83.3 and 91.3%, respectively (Fig. 6).

## Discussion

In comparison with previous studies, there are similarities and differences in the CT findings of benign and malignant patchy GGOs and GGNs. In the present study, patients with benign and malignant patchy GGOs differed in age, gender, and history of cancer, and the lesions were also different in their CT findings. Specifically, it was found that patients with malignant GGOs were more likely to be older, female, have a lower incidence of malignant tumor history; lesions located in the upper lobe and distributed along the bronchovascular bundles, lesions with centric solid component, dilated internal bronchus, and lesions with well-defined boundary were more closely associated with malignant patchy GGOs.

Numerous studies discovered that being female and old were significantly associated with malignant pulmonary nodules, especially those who presented with GGNs [9, 23–29]. In this study, malignant GGOs were also more frequently found in old and female patients. A heavy smoking history was a key factor for risk assessment in lung cancer screening criteria [30]. A previous study reported that a higher smoking index was associated with malignant GGNs [31]. However, in this study, no difference in smoking history was found between patients with benign and malignant GGOs. It is slightly surprising that the incidence of a malignant tumor history was higher in benign GGOs, possibly due to the reduced immunity of patients with tumors, making them more susceptible to pulmonary infections.

Previous studies found that the lesion size was a good indicator of malignant GGNs or invasiveness [1, 19, 20, 23, 31–33]. Smaller nodules are commonly benign, while larger ones tend to be malignant [1, 19, 23, 31]. With an increasing size, the invasiveness of lung adenocarcinoma with GGN also increased accordingly; the GGN size could help differentiate pre-invasive from invasive lesions [20, 23, 32, 33]. In this study, the size of lesions tended to increase from AAH to IAC. However, no significant difference in size was observed between benign and malignant GGOs. Thus, the size may not be an effective indicator for predicting the nature of patchy GGOs.

Compared with pGGNs, mGGNs had a higher possibility of malignancy or invasiveness [9, 10, 15, 17, 19, 32, 34–36]. More IAC nodules manifested as mGGNs [10, 15, 32, 35]. In this study, a correlation between GGO density and malignancy was also observed, mGGOs had a higher possibility to be malignant than pGGOs. Moreover, among mGGOs, those with centric solid component were more likely to be malignant. This finding was consistent with the result of a previous study on GGNs [37]. Pathologically, peripheral GGOs are caused by a local tumor spread or so-called lepidic growth pattern in which tumor cells proliferate along the surface of intact alveolar walls without interstitial or vascular invasion [38].

The location of the lesions can also contribute in differentiating malignant from benign GGNs [31]. Lung cancer was more common in the upper lobe [39]. In this study, malignant GGOs were also more frequently located in the upper lobe. Therefore, patchy GGOs located in the upper lobe should receive more attention, and it is necessary to evaluate whether these are neoplastic lesions.

The dilated bronchus sign caused by the retraction of tumor fibrosis can also be an independent predictor for malignant GGNs [40, 41]. In this study, the dilated internal bronchus was more frequent in malignant GGOs than in benign ones. However, a previous study showed that this sign had no significant difference between malignant GGNs and benign GGNs [42]. This might be explained by the very few numbers of nodules with dilated bronchus in their study. Therefore, internal bronchial changes should be considered in the differentiation of GGOs.

The boundary was another valuable indicator of malignant GGNs or invasiveness [1, 6, 16, 17, 33]. Most studies reported that a well-defined GGN was more likely to be malignant [1, 6, 17]. Wu et al. [16] found that the occurrence of a clear tumor-lung interface tended to increase from AAH to IA. Because of the increase in invasiveness degree, the tumor cell arrangement on alveolar walls became dense and thickened [6]. In contrast, cellular infiltrates in benign GGNs were mainly inflammatory cells, and they might gradually diminish and subsequently cause an indistinct border. The CT manifestation of benign and malignant patchy GGOs in the boundary is in accordance with that of GGNs, so it is important to evaluate their boundary.

In addition to the above features, the relationship between the lesions and bronchovascular bundles was another crucial indicator in discriminating benign from malignant GGOs. This was not noted in previous studies about GGNs. Malignant GGOs frequently distributed along the bronchovascular bundles, while benign GGOs often crossed the bronchovascular bundles, distributed among the bronchovascular bundles, or in the subpleural zone. Thus, an accurate evaluation of the lesion distribution in relation to the bronchovascular bundles is very important. In this study, MPR images enabled the bronchus and blood vessels to be demonstrated continuously and wholly; thus, the relationship between the largest section of lesions and the long axis of bronchovascular bundles can be well displayed and evaluated.

This study has two limitations and further research is needed. First, this is a retrospective study, and the data were from a single institution. Therefore, there may have been a selection bias. Further prospective and multi-center studies may provide more accurate results. Second, although this study included benign GGOs diagnosed clinically by interval shrinkage or disappearance, benign GGOs may not change in size even after a long follow-up period. Such lesions might have distinctive features but were not incorporated in this study. Therefore, the discriminating CT findings of such lesions from malignant ones were not revealed in this study.

## Conclusions

There are many differences between benign and malignant patchy GGOs based on their clinical characteristics and CT features. These patients differed in age, gender, and history of cancer, while the lesions differed in the location, distribution in relation to the bronchovascular bundles, density pattern, internal bronchial changes, and boundary. The application of MPR images to evaluate the relationship between the lesions and bronchovascular bundles is of great significance in differentiating them. In older patients (≥58 years), well-defined patchy GGOs with centric solid component, locating in the upper lobe, and distributing along the bronchovascular bundles are highly likely to be malignant.

## Data Availability

The datasets used and/or analysed during the current study are not publicly available because the cases are from the Picture Archiving and Communicating System of our Hospital but are available from the corresponding author on reasonable request.

## Abbreviations

CT: Computed tomography
GGO: Ground-glass opacity
HRCT: High-resolution computed tomography
mGGO: Mixed ground-glass opacity
pGGO: Pure ground-glass opacity
GGN: Ground-glass nodule
OR: Odds ratio
CI: Confidence interval
MPR: Multiplanar reconstruction
ROC: Receiver operator characteristic
AAH: Atypical adenomatous hyperplasia
AIS: Adenocarcinoma in situ
MIA: Minimally invasive adenocarcinoma
IAC: Invasive adenocarcinoma
